# Survival and re-aerosolization in dust of Mycobacterium smegmatis, a surrogate marker for Mycobacterium tuberculosis

**DOI:** 10.1186/2047-2994-4-S1-P100

**Published:** 2015-06-16

**Authors:** KV Tshilombo, S Mehtar, S Sampson, R Warren, NL Steyn

**Affiliations:** 1 Interdisciplinary Health Sciences: Infection Prevention and Control, Faculty of Health Sciences, University of Stellenbosch, Cape Town, South Africa; 2 Laboratory of the National Research Foundation Centre of Excellence for Tuberculosis Research/Tygerberg campus, University of Stellenbosch, Cape Town, South Africa

## Introduction

*Mycobacterium tuberculosis* (MTB), an essentially airborne pathogen transmitted via aerosols remains viable while in the soil, outside its hosts for extended periods of time. It has been suggested that MTB cannot be re-aerosolized and cause disease once it has landed outside the body in dust or the environment.

## Objectives

This project aimed to answer several questions relating to MTB and its ability to cause disease after re-aerosolization: Can MTB be re-aerosolised? Does re-aerosolized MTB remain viable? If viable, can it cause infection? Can environmental bioburden be reduced using copper surfaces?

## Methods

This prospective *in vitro* study was of two phases (1^st^non-copper and 2^nd^copper) preceded by a pilot study. Non-pathogenic fast growing *Mycobacterium smegmatis*(MSM) was used as a surrogate for MTB. 125 mg of sterile dust was spread in a sealed plexiglass aerosol chamber prior to nebulization of 20 ml of 10^6^ cfu/ml of MSM.A six stage Anderson air sampler and settle plates were combined for sampling before and after re-aerosolization of dust using two small fans. Plates incubation at 37ºC lasted 3-10 days. Estimation of CFU number was based on viable plate count.

## Results

MSM survived for more than 19 days in dust in the absence of copper and could be re-aerosolized, whereas in the presence of copper, its survival rate was about 10 days after nebulization in dust- almost 50% less than on a non-copper surface. Twenty-four hours after nebulization, was noted a significant decrease in both copper (17.88%) and non-copper (100%) for respirable particles, but copper still showed significantly lower levels of mycobacteria.

## Conclusion

MSM, surrogate marker for MTB survived in dust and remained viable after re-aerosolization. This study demonstrated that mycobacteria can be re-aerosolized and remains viable. This is particularly relevant in low to middle income countries with high MTB bioburden where dust is common and sweeping in healthcare facilities is frequent. It also illustrated that anti-microbial properties of copper surface remain effective in dust. Copper can be used as a touch surface to reduce the bioburden of microbes including mycobacteria.

## Disclosure of interest

None declared.

